# Reliability of an e-PRO Tool of EORTC QLQ-C30 for Measurement of Health-Related Quality of Life in Patients With Breast Cancer: Prospective Randomized Trial

**DOI:** 10.2196/jmir.8210

**Published:** 2017-09-14

**Authors:** Markus Wallwiener, Lina Matthies, Elisabeth Simoes, Lucia Keilmann, Andreas D Hartkopf, Alexander N Sokolov, Christina B Walter, Nina Sickenberger, Stephanie Wallwiener, Manuel Feisst, Paul Gass, Peter A Fasching, Michael P Lux, Diethelm Wallwiener, Florin-Andrei Taran, Joachim Rom, Andreas Schneeweiss, Joachim Graf, Sara Y Brucker

**Affiliations:** ^1^ Gynecologic Oncology, National Center for Tumor Diseases, Hospital for General Obstetrics and Gynecology University Hospital Heidelberg Heidelberg Germany; ^2^ Research Institute for Women’s Health Department of Women’s Health University Hospital Tuebingen Tuebingen Germany; ^3^ Department of Women’s Health University Hospital Tuebingen Tuebingen Germany; ^4^ Institute for Medical Biometry and Informatics University Hospital Heidelberg Heidelberg Germany; ^5^ Department of Gynecology and Obstetrics University Hospital Erlangen Erlangen Germany

**Keywords:** breast cancer, patient-reported outcomes, HRQoL, EORTC QLQ-C30, reliability

## Abstract

**Background:**

Breast cancer represents the most common malignant disease in women worldwide. As currently systematic palliative treatment only has a limited effect on survival rates, the concept of health-related quality of life (HRQoL) is gaining more and more importance in the therapy setting of metastatic breast cancer. One of the major patient-reported outcomes (PROs) for measuring HRQoL in patients with breast cancer is provided by the European Organization for Research and Treatment of Cancer (EORTC). Currently, paper-based surveys still predominate, as only a few reliable and validated electronic-based questionnaires are available. Facing the possibilities associated with evolving digitalization in medicine, validation of electronic versions of well-established PRO is essential in order to contribute to comprehensive and holistic oncological care and to ensure high quality in cancer research.

**Objective:**

The aim of this study was to analyze the reliability of a tablet-based measuring application for EORTC QLQ-C30 in German language in patients with adjuvant and (curative) metastatic breast cancer.

**Methods:**

Paper- and tablet-based questionnaires were completed by a total of 106 female patients with adjuvant and metastatic breast cancer recruited as part of the e-PROCOM study. All patients were required to complete the electronic- (e-PRO) and paper-based versions of the HRQoL EORTC QLQ-C30 questionnaire. A frequency analysis was performed to determine descriptive sociodemographic characteristics. Both dimensions of reliability (parallel forms reliability [Wilcoxon test] and test of internal consistency [Spearman rho and agreement rates for single items, Pearson correlation and Kendall tau for each scale]) were analyzed.

**Results:**

High correlations were shown for both dimensions of reliability (parallel forms reliability and internal consistency) in the patient’s response behavior between paper- and electronic-based questionnaires. Regarding the test of parallel forms reliability, no significant differences were found in 27 of 30 single items and in 14 of 15 scales, whereas a statistically significant correlation in the test of consistency was found in all 30 single items and all 15 scales.

**Conclusions:**

The evaluated e-PRO version of the EORTC QLQ-C30 is reliable for patients with both adjuvant and metastatic breast cancer, showing a high correlation in almost all questions (and in many scales). Thus, we conclude that the validated paper-based PRO assessment and the e-PRO tool are equally valid. However, the reliability should also be analyzed in other prospective trials to ensure that usability is reliable in all patient groups.

**Trial Registration:**

ClinicalTrials.gov NCT03132506; https://clinicaltrials.gov/ct2/show/NCT03132506 (Archived by WebCite at http://www.webcitation.org/6tRcgQuou).

## Introduction

### Epidemiological Relevance of Breast Cancer

Breast cancer represents the most common malignant disease in women worldwide, with more than 71,000 new cases diagnosed every year in Germany [1]. In spite of improvement in progression-free survival (PFS) through promising targeted therapy options for hormone receptor-positive breast cancer, metastatic breast cancer remains an incurable disease [2-7]. In the year 2012, 17,853 women died of breast cancer in Germany alone [1]. Although early-stage breast cancer is often associated with high survival rates, the prognosis of metastatic breast cancer is significantly poorer, and therefore, the aim of treatment is mostly palliative because of minor probability of curation in patients with metastatic breast cancer [8]. Depending on the phenotype, median overall survival (OAS) after diagnosis of metastatic breast cancer is 2-3 years [9], ranging from 13.3 months for triple-negative [10] and 34.4 months for HER2-positive subtype of breast cancer [11].

### Health-Related Quality of Life in Metastatic Breast Cancer

As currently systematic palliative treatment had only limited effect on survival rates, the concept of health-related quality of life (HRQoL) is gaining more and more importance in the therapy setting of metastatic breast cancer. Especially against the background of emerging side effects accompanying multiple oncological treatment lines, treatment should primarily aim at restoration and conservation of patients’ HRQoL before prolonging the survival of patients [12]. Additionally, the diagnosis of an incurable disease represents an enormous emotional burden resulting in psychosocial distress that might impair the patient’s well-being [1,13,14]. This is also taken into account by the recent German S3-guidelines for diagnosis, treatment, and aftercare of patients with breast cancer, recommending regular assessment of HRQoL during treatment [15]. HRQoL is defined as an *individual’s perception of their position in life in the context of the culture and value systems in which they live and in relation to their goals, expectations, standards, and concerns* by World Health Organization [16]. HRQoL is divided into domains (1) health, (2) subjective feelings, (3) leisure time activities, (4) social relationships, (5) general activities, and (6) life satisfaction [17]. For many years, HRQoL in patients with breast cancer has been investigated. Especially in patients with metastatic disease, the measurement of HRQoL is important, as the primary goal of therapy is to afford them a high quality of life during their remaining lifespan [18-20].

### Health-Related Quality of Life and Patient-Reported Outcomes

The most important obstacle has been the absence of widely accepted, standardized methods for carrying out such assessments, as much of the data suggest that clinicians miss or underestimate a large proportion of the symptomatic adverse events experienced by patients [21,22]. Furthermore, the assessment of adverse events and HRQoL by health care professionals is inconsistent when compared with the opinion of other professionals [23-25]. In this context, an independent report by the patient herself through patient-reported outcomes (PROs) could be more reliable and feasible. The US Food and Drug Administration (FDA) defines a PRO as *any report of the status of a patient’s health condition that comes directly from the patient (or in some cases, a caregiver or surrogate), without interpretation of the patient’s response by a clinician or anyone else* [26]. PROs comprise various aspects of the subjectively perceived state of health from patient’s point of view, such as HRQoL, satisfaction with care, and drug adherence [27-29]. In this closely related areas, validated PROs are already the accepted gold standard for data collection, being used in clinical trials, as well as regulatory drug approvals (approximately 25% of US drug labels now include PRO-derived data) [30-33]. This aspect is also designated for optimization within the National Cancer Plan to enable information exchange between in-patient and outpatient treatment [30]. Concerning feasibility of patient’s self-reported state of health in the oncological setting, a previous study demonstrated that most patients are willing and able to self-report their experiences with treatment [34]. Moreover, the Eastern Cooperative Oncology Group could not find any association between the patient’s performance or functional status and compliance rates [30,35]. A study indicated that integrating HRQoL reports in daily clinical routine might represent an effective and time-saving option to improve medical care, as communication between patient and physician can be facilitated without extended interviews with the patient [36]. However, although regular assessment of HRQoL is generally recommended, routine evaluation is not yet provided in clinical practice [15,37]. One of the major PROs for measurement HRQoL in patients with breast cancer is provided by the European Organization for Research and Treatment of Cancer (EORTC). The EORTC QLQ-C30 as a modular approach is available in more than 100 languages and is used to assess HRQoL in patients with various cancers within the scope of clinical trials, as well as in daily routine [38].

### Electronic Monitoring of Patient-Related Outcome

With the expansion of digital tools, assessment of PRO by using an electronic equipment (e-PRO), such as tablet computers, is becoming a promising and economically viable approach, as real-time HRQoL monitoring allows early detection of patients at risk, ongoing improvement of oncological treatment, and ensuring the patient’s safety [12]. Current data show that monthly compliance referring to frequency and completion of questionnaires with home Web reporting was high in breast cancer, warranting strategies to enhance compliance with routine care settings [30]. Additionally, some studies have suggested high feasibility of an electronic patient self-report platform in oncological patients, with mean compliance rates ranging from 75% to 85%, high patient satisfaction, and good usability of systems even among the non-Web avid and elderly patients [39,40]. Velikova et al [36] demonstrated that routine electronic HRQoL assessment in patients with breast cancer could positively influence physician-patient communication, potentially improving emotional functioning and HRQoL. Additionally, there is evidence that the completion of the questionnaire itself may improve the patient’s well-being, regardless of whether the results are fed back to physicians [36]. However, knowledge regarding patient acceptance, feasibility, and barriers remains limited, especially in relation to health status, socioeconomic aspects, and the influence of other variables on patient’s response behavior [41-44]. Currently, paper-based surveys of PRO still predominate, as there are only a few reliable and validated e-PRO questionnaires. The paper-based versions are frequently assigned into a tablet-based format without verification of reliability. As the aspects that influence the patient’s willingness to use e-PRO and their response behavior by using e-PRO remain unclear, this strategy can endanger significance of e-PRO surveys [41]. For instance, the EORTC QLQ-C30 questionnaire has been used worldwide [45], but only reliable paper-based versions of it, although e-PRO has become much more prevalent (and “user-friendly”) [46]. Facing the possibilities that are coming along with the evolving digitalization in medicine, the validation of electronic versions of well-established PRO is essential in order to contribute to a comprehensive and holistic oncological care and to ensure high quality in cancer research.

### Aims and Objectives

The aim of this study was to analyze the reliability of a tablet-based e­–PRO-measuring application for EORTC QLQ-C30 in German in patients with adjuvant (curative) and metastatic breast cancer compared with the established paper-based version. It should be analyzed if the response behavior of patients with breast cancer is influenced by the type of answering the questionnaire (answering by using paper and pencil or tablet computer) in a statistically significant way. We wanted to know whether there are differences in response behavior between the validated paper-based PRO version of EORTC QLQ-C30 and a new e-PRO version. The other aim was to examine the feasibility of using an e-PRO version of the EORTC QLQ-C30 for the future tablet-based measurement of HRQoL in patients with metastatic and adjuvant breast cancer in clinical practice. To achieve the aims, the patients were asked to fill out both paper- and tablet-based EORTC QLQ-C30 questionnaires.

## Methods

### Sample and Study Design

From July 2015 to May 2016, paper- and tablet-based PRO questionnaires were completed by a total 106 female patients with adjuvant and metastatic breast cancer treated consecutively at the Department of Women’s Health in Tuebingen, Germany, and the National Cancer Centre in Heidelberg, Germany. The patients were recruited as part of the e-patient-reported outcomes and compliance analysis (PROCOM) study. The aim of e-PROCOM was to evaluate the general patient acceptance and practicability of a Web-based application for a PRO questionnaire for patients with adjuvant or metastatic breast cancer. The patients were asked to participate to compare the response behavior in paper- and Web-based questionnaires for analyzing the reliability of the e-PRO versions of the EORTC QLQ-C30 questionnaires (version 3) [38]. Inclusion criteria of e-PROCOM were female gender, full legal age (18 years and older), adjuvant or metastatic breast cancer diagnosis, sufficient language skills in German, and signed declaration on consent forms. Exclusion criterion was participation in other studies to minimize the burden of questionnaires. The patients were asked to complete the questionnaire during an outpatient visit to the hospital under the supervision of an attending physician. The study was designed as a double-centered (Tuebingen and Heidelberg), two-armed, prospective randomized trial. All patients were required to fill out the electronic- (e-PRO) and paper-based HRQoL questionnaire of EORTC QLQ-C30. Patients in arm A were assigned tablet computer, followed by paper questionnaire in the same session. Patients in arm B filled out the paper-based version, followed by the tablet-based questionnaire. The randomization procedure is based on the permuted-block randomization, which strives to generate equally large groups of treatment [47,48]. The postexposure acceptance for using the e-PRO tool was high (92%), as the patients were asked whether they could potentially imagine using tablet-based tools before using e-PRO [42]. Patients were informed about the aims of the study and asked for their consent ex ante. The study was approved by the Ethics Committee at the University of Tuebingen (project number 089/2015B02).

### Procedure

The data collection was performed in 5 parts. The first part focused on the patientsʼ socio-economic variables. The second part contained the EORTC QLQ-C30, consisting of 30 questions in 5 subscales, various symptom scales, and individual items related to the patientsʼ health status on a multidimensional level. In addition, 28 of 30 questions are designed with a 4-point Likert scale and 2 questions with a 7-point Likert scale. Mean values were calculated in accordance with the official EORTC guidelines, which require a separate score to be calculated for each scale. The scores range from 0 to 100 [38,49]. The third part of the assessment also targeted HRQoL by administering FACT-B questionnaire, consisting of 37 questions with responses in 5 dimensions on a 5-point Likert scale [50,51]. The publication of FACT-B’s e-PRO reliability analysis is under preparation. The patients in the fourth part of the assessment were asked about preexisting technical skills, their willingness to use e-PRO, and potential barriers in relation to their health status [42], and the fifth part concerned the patients’ evaluation of the e-PRO tool (publication in preparation). The patients filled out the second and third parts of the assessment both paper- and tablet-based, whereas they answered only paper-based questionnaire in the other parts. In this study, we report the results of the second part of the assessment (reliability analyses of e-PRO tool of EORTC QLQ-C30).

### Specifics of the PRO Tool

**Figure 1:**
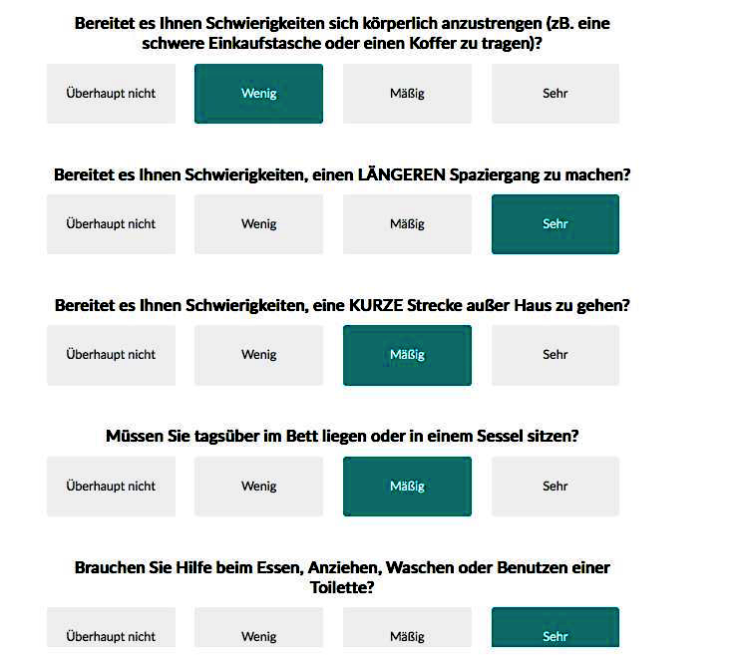
Screenshot of PiiA application of the EORTC QLQ-C30 questionnaire for 4-point-scale questions (German).

**Figure 2:**
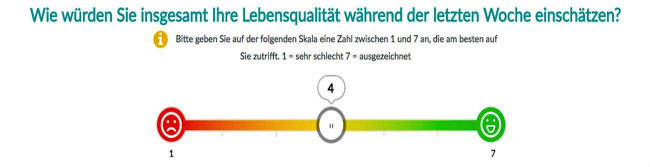
Screenshot of PiiA application of the EORTC QLQ-C30 questionnaire for 7-point-scalekaled questions (German).

For e-PRO measurement, we used the PiiA (“Patient-informiert-interaktiv-Arzt”) Web-based application, which presents the relevant questions to be completed on a tablet computer. The PiiA-portal is a Web-based solution for capturing PROs, which was self-developed by the working group. Patients receive anonymous user credentials and are asked to complete FACT-B and QLQ-C30 questionnaires. Figure 1 shows the user interface of the first set of questions of the German EORTC QLQ-C30. The tool is constructed similar for all 28 questions with a 4-point Likert scale. Figure 2 shows the user interface of the 7-point Likert scale questions. After completing the questionnaires, patients log out and the pseudo-anonymized data will be backed up on a local storage device and securely locked.

### Statistical Analyses

**Figure 3:**
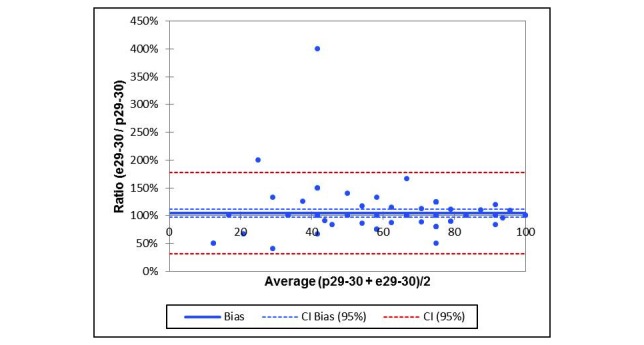
Bland-Altman plot for overall state of health.

A frequency analysis was first performed using IBM SPSS Statistics (version 24) to determine the descriptive sociodemographic characteristics of the patients. After that, we analyzed both dimensions of reliability (parallel forms reliability and test of internal consistency) and examined the disparity of responses and the rate of consistency between the paper-based PRO and e-PRO answers. Both dimensions of reliability were calculated for the 30 single items and 15 scales, resulting from the single items in accordance with the EORTC guidelines [49]. According to the Shapiro-Wilks test, the paired samples were not normally distributed, and therefore, we used the Wilcoxon test to identify possible statistically significant differences in the test of parallel forms reliability. Additionally, a Bland-Altman plot was created for the scale “overall state of health,” which represents the HRQoL (Figure 3). Earlier, the mean values of the paper-based PRO and the e-PRO measurements were calculated with the official EORTC guidelines [49], which require a separate score calculated for each scale, with scores ranging between 0 and 100. The consistency analyses were performed by calculation of correlation analyses (Spearman rho and agreement rates) for every EORTC question together with inter-item correlation (Pearson correlation) and rank correlation (Kendall tau) for each scale. With Pearson correlation, the internal consistency of a scale can be measured; it describes the extent to which the tasks or questions of a scale are interrelated. While Spearman rho test examines the internal consistency of the individual questions (specifically, the reliability of the e-PRO individual questions against the paper-based questions), Pearson correlation and Kendall tau tests are used to determine the scales calculated according to EORTC guidelines. Previously, we performed chi-square and Shapiro-Wilks tests between patients with metastatic and adjuvant breast cancer to identify possible statistically significant differences in relation to HRQoL. In all analyses, *P* values <.05 (2-tailed) were considered indicative of statistically significant differences (α=.05). As the analysis behaves as an explorative study, all reported *P* values can be received as purely descriptive. As we did not find any significant differences in relation to the response behavior between arms A and B in a pretest, we assessed both the arms together to compare the paper-based and e-PRO questionnaires of the patients. Bland-Altman plot was produced by using XLSTAT 2017. Missing values (which arose when patients did not answer individual questions) were ignored in the statistical calculation.

## Results

### Patient Enrollment

**Figure 4:**
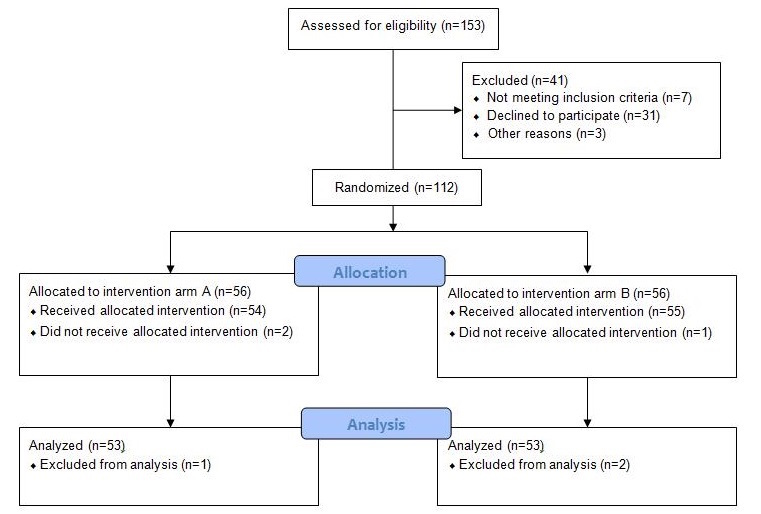
The CONSORT flow diagram.

Overall, 106 female patients with breast cancer were recruited, who completed both paper- and tablet-based EORTC QLQ-C30 questionnaires. Originally, n=153 patients were assessed for eligibility, of which 47 were excluded during recruiting, allocation, and data analyses as shown in the Consolidated Standards of Reporting Trials (CONSORT) flow diagram (Figure 4). A total of 53 patients were assigned tablet computer, followed by paper in the same session (arm A), whereas the same number of patients filled out the paper-based version, followed by the tablet-based questionnaire (arm B). Patients who had not completed more than half of the EORTC questions either paper- or tablet-based were excluded (1 patient in arm A and 2 patients in arm B). We did not find significant differences between the two arms in the response behavior likewise in sociodemographic status and in therapy setting, wherefore both arms were appreciated together. Previously, both arms were compared in all single items. Furthermore, 10 patients (arm A) and 16 patients (arm B), respectively, produced missing data in some questions (more often in the tablet-based questionnaire).

### Sociodemographic Variables

Tables 1 and 2 show the sociodemographic characteristics of the study group, with 72% patients in adjuvant therapy and 28% in metastatic situation. We did not find significant intragroup differences between patients with metastatic and adjuvant breast cancer either in e-PRO or in paper-based PRO. Although the adjuvant and metastatic patients differ by focalizing age and HRQoL as the metastatic patients were older and reported a poorer HRQoL, we found no differences between the e-PRO response behavior of both groups. There were no differences in reliability of all single items and scales between metastatic and adjuvant patients because of which the whole collective was appreciated together. The mean age of the whole collective amounted to 51.0 years, and nearly one-third of the patients showed a higher level of education (high school diploma).

**Table 1:** Sociodemographic characteristics of the patients, part I.

Sociodemographic characteristics		Descriptive analyses (N=106)
**Age**		
	Mean (SD)	51.0 (11.31)
	Median (range, minimum-maximum)	52 (54, 30-84)
**Level of education (1=lowest; 5=highest)**		
	Median	3.0
	Interquartile range (25%-Quartil; 75%-Quartil)	2.0 (3.0; 5.0)

**Table 2:** Sociodemographic characteristics of the patients, part II.

Sociodemographic characteristics		n (%)	95% CI
**Kind of education**			
	No qualification	1 (.9)	0-6
	Main or secondary school leaving certificate	43 (40.6)	32-50
	Advanced technical certificate	19 (17.9)	10-26
	High school diploma (“Abitur”)	33 (31.1)	22-40
	Not specified	10 (9.4)	2-15
**Therapy setting**			
	Metastatic	30 (28.3)	19-35
	Adjuvant treatment	76 (71.7)	61-83

### Parallel Forms Reliability

The e-PRO tool seems to be reliable in the dimension of parallel forms reliability, as only few significant differences could be found. Table 3 shows the results of the Wilcoxon test of the 30 single items in the EORTC QLQ-C30. In only 3 items (in relation to *tiredness* and *pain* and *need to rest*), there were weak statistically significant differences between paper-based PRO and e-PRO. Tiredness was ranked a bit higher in the e-PRO questionnaire with noticeable differences by focalizing median’s characteristics (MD_Paper-based PRO_=2.0 vs MD_e-PRO_=3.0) similar to need to rest, whereas the patients confirmed more pain in the paper-based questionnaire. In 27 of the questions in the EORC QLQ-C30, there were no statistically significant differences between the patient’s answers in the paper-based questionnaire and e-PRO.

No statistically significant differences could be found in the Wilcoxon test for scoring values in the function and symptom scales of the EORTC QLQ-C30, except for the score “tiredness,” where the difference was significantly low (Table 4). The patient’s response behavior between paper-based PRO and e-PRO was similar on the 5 functional scales, the 9 symptom scales, and in the overall state of health, as the identified differences in the single items had balanced each other in relation to the scoring values’ calculation.

Figure 3 shows a Bland-Altman plot of the scale “overall of health” to compare agreement of measurements in a graphical way by plotting the difference between paper-based PRO and e-PRO against their mean. It is apparent that the deviations from the mean value of the difference are almost exclusively within the confidence interval, so the response behavior between paper-based PRO and e-PRO is not significantly different in the collective.

**Table 3:** Parallel forms reliability (Wilcoxon test) in single items.

Scale no.	Item no.	Short description of item	Paper-based PRO^a^		Electronic-based PRO		*P* value
			Mean (SD)	Median (interquartile range)	Mean (SD)	Median (interquartile range)	
I	1	Strenuous activities	2.40 (1.13)	2.0 (2.0)	2.33 (1.11)	2.0 (2.0)	.18
I	2	Long walk	2.19 (1.10)	2.0 (2.0)	2.23 (1.13)	2.0 (2.0)	.79
I	3	Short walk	1.56 (.85)	1.0 (1.0)	1.58 (.86)	1.0 (1.0)	>.99
I	4	Stay in bed or chair	1.79 (.87)	2.0 (1.0)	1.75 (.79)	2.0 (1.0)	.29
I	5	Self-care	1.18 (.55)	1.0 (.0)	1.14 (.51)	1.0 (0.0)	>.99
II	6	Limited in work	2.22 (1.05)	2.0 (2.0)	2.28 (.99)	2.0 (1.0)	.19
II	7	Hobbies or limited leisure activities	2.30 (1.10)	2.0 (2.0)	2.38 (1.04)	2.0 (1.0)	.18
III	21	Tense	2.06 (.85)	2.0 (2.0)	2.06 (.80)	2.0 (2.0)	.82
III	22	Worried	2.40 (.90)	2.0 (1.0)	2.49 (.81)	3.0 (1.0)	.31
III	23	Irritated	2.01 (.78)	2.0 (2.0)	2.02 (.79)	2.0 (2.0)	.71
III	24	Depressed	2.12 (.90)	2.0 (2.0)	2.18 (.91)	2.0 (2.0)	.85
IV	20	Concentration	1.86 (.97)	2.0 (1.0)	1.95 (1.06)	2.0 (2.0)	.06
IV	25	Memory difficulties	1.93 (.90)	2.0 (2.0)	1.96 (.91)	2.0 (2.0)	.53
V	26	Family life	2.21 (.96)	2.0 (2.0)	2.36 (1.01)	2.0 (1.0)	.09
V	27	Social life	2.39 (1.02)	2.0 (1.0)	2.41 (1.05)	2.0 (1.0)	.64
VI	10	Need to rest	2.33 (.93)	2.0 (1.0)	2.48 (.88)	2.0 (1.0)	*.005*^b^
VI	12	Felt weak	2.26 (.99)	2.0 (1.0)	2.20 (1.02)	2.0 (2.0)	.59
VI	18	Felt tired	2.48 (.93)	2.0 (1.0)	2.55 (.89)	3.0 (1.0)	*.03*^b^
VII	14	Nausea	1.54 (.78)	1.0 (1.0)	1.55 (.80)	1.0 (1.0)	.95
VII	15	Vomiting	1.09 (.45)	1.0 (.0)	1.13 (.54)	1.0 (.0)	>.99
VIII	9	Had pain	1.94 (.93)	2.0 (2.0)	1.92 (.85)	2.0 (2.0)	*.02*^b^
VIII	19	Pain interfered	1.95 (.99)	2.0 (2.0)	1.91 (.93)	2.0 (1.5)	.19
IX	8	Shortness of breath	1.92 (.87)	2.0 (1.0)	1.84 (.87)	2.0 (1.0)	.27
X	11	Sleep disturbance	2.15 (1.01)	2.0 (2.0)	2.23 (1.05)	2.0 (2.0)	.13
XI	13	Lack of appetite	1.56 (.88)	1.0 (1.0)	1.54 (.87)	1.0 (1.0)	.89
XII	16	Constipation	1.52 (.78)	1.0 (1.0)	1.47 (.70)	1.0 (2.0)	.95
XIII	17	Diarrhea	1.43 (.76)	1.0 (1.0)	1.40 (.72)	1.0 (1.0)	.15
XIV	28	Financial impact of disease	1.97 (1.03)	2.0 (2.0)	1.93 (1.03)	2.0 (2.0)	.28
XV	29	Physical condition	4.63 (1.43)	5.0 (2.0)	4.63 (1.37)	5.0 (2.0)	.95
XV	30	General QoL^c^	4.67 (1.49)	5.0 (2.13)	4.72 (1.59)	5.0 (2.0)	.29

^a^PRO: Patient reported outcomes.

^b^Statistically weak significant difference.

^c^QoL: quality of life.

**Table 4:** Parallel forms reliability (Wilcoxon test) for scoring values in the function and symptom scales.

Scale		Number of questions (items) QLQ‑C30	Paper-based PRO^a^		Electronic-based PRO		*P* value	
			Mean (SD)	Median (interquartile range)	Mean (SD)	Median (interquartile range)		
**Functional scales**							
	Physical resilience^a^ (I)	5 (1-5)	73.14 (24.83)	80.0 (33.3)	73.89 (23.65)	80.0 (33.3)		.08
	Resilience at work and during leisure time activities^a^ (II)	2 (6-7)	57.92 (34.61)	66.67 (50)	55.77 (32.41)	66.67 (50)		.59
	Emotional resilience^a^ (III)	4 (21-24)	61.53 (23.95)	66.67 (41.67)	60.88 (23.22)	58.33 (33.34)		.53
	Cognitive resilience^a^ (IV)	2 (20-25)	70.18 (26.00)	66.67 (50)	68.67 (27.67)	66.67 (50)		.12
	Social resilience^a^ (V)	2 (26,27)	56.93 (30.46)	66.67 (50)	54.23 (32.58)	66.67 (50)		.12
**Symptom scales**							
	Tiredness (VI)	3 (10,12,18)	45.21 (28.41)	33.33 (36.11)	45.10 (28.08)	33.33 (44.44)		*.05*^b^
	Nausea or vomiting (VII)	2 (14,15)	10.42 (18.13)	.0 (1.47)	11.52 (19.80)	.0 (3.19)		.28
	Pain (VIII)	2 (9,19)	31.25 (30.46)	33.33 (50.0)	29.34 (26.65)	33.33 (50.0)		.35
	Shortness of breath (IX)	1 (8)	30.58 (29.06)	33.33 (33.33)	28.00 (29.10)	33.33 (33.33)		.70
	Sleep disturbance (X)	1 (11)	38.45 (33.64)	33.33 (66.67)	41.06 (34.84)	33.33 (66.67)		.71
	Lack of appetite (XI)	1 (13)	18.65 (29.18)	.0 (33.33)	18.14 (29.04)	.0 (33.3)		.72
	Constipation (XII)	1 (16)	17.31 (25.87)	.0 (33.33)	15.69 (23.37)	.0 (33.33)		.47
	Diarrhea (XIII)	1 (17)	14.22 (25.41)	.0 (33.33)	13.27 (23.96)	.0 (33.33)		.19
	Financial impact of disease (XIV)	1 (28)	32.19 (34.33)	33.33 (66.67)	31.05 (34.40)	33.33 (66.67)		.20
	Overall state of health^a^ (XV)	2 (29,30)	60.78 (23.75)	66.67 (41.67)	61.30 (23.82)	66.67 (33.33)		.52

^a^Items are scaled from worst to best, with high scores representing a good QoL profile.

^b^Statistically weak significant difference.

### Test of Internal Consistency

Table 5 shows the Spearman rho correlation values and agreement rates, which were obtained for every question of the EORTC QLQ-C30 questionnaire. In all 30 questions, a high correlation (>.7) was found between paper-based PRO and e-PRO. In 23 questions, the correlation levels was >.85, and in 1 question, we found a maximal correlation of 1.0. In all 30 correlated questions, agreement rates fluctuated between 66.6% and 100%.

Table 6 shows the results of internal consistency testing for the function and the symptom scales of the EORTC between paper-based PRO and e-PRO. There were high correlations in the response behavior (>.7) in all 5 functional scales, all 9 symptom scales, and the overall state of health. Statistically high significant correlations (>.9) between paper-based PRO and e-PRO was found in all 5 functional scales by focalizing interitem correlation (Pearson correlation). The rank correlation in all functional scales was also high, as Kendall tau coefficient ranged between .79 and .92. In the overall state of health, the correlation was .88 (Pearson correlation) and .77 (Kendall tau). In the symptom scales, the consistency rates were between .80 (*shortness of breath*) and .96 (*lack of appetite*) in relation to interitem correlation, together with rates between .71 (*shortness of breath*) and .93 (*diarrhea*) with regard to rank correlation.

**Table 5:** Test of internal consistency in single items: results of correlation (Spearman rho) and agreement analysis.

Scale no.	Item no.	Short description of items	Spearman rho	*P* value of Spearman rho^b^	Agreement (%)
I	1	Strenuous activities	.932	<.001	81.4
I	2	Long walk	.949	<.001	86.6
I	3	Short walk	.930	<.001	91.8
I	4	Stay in bed or chair	.848	<.001	84.1
I	5	Self-care	.999	<.001	97.4
II	6	Limited in work	.863	<.001	76.8
II	7	Hobbies or limited leisure activities	.867	<.001	81.8
III	21	Tense	.936	<.001	74.3
III	22	Worried	.846	<.001	80.6
III	23	Irritated	.945	<.001	90.2
III	24	Depressed	.812	<.001	77.2
IV	20	Concentration	.900	<.001	79.5
IV	25	Memory difficulties	.936	<.001	86.2
V	26	Family life	.876	<.001	75.3
V	27	Social life	.891	<.001	78.9
VI	10	Need to rest	.860	<.001	78.2
VI	12	Felt weak	.878	<.001	85.3
VI	18	Felt tired	.829	<.001	78.8
VII	14	Nausea	.924	<.001	89.4
VII	15	Vomiting	1.00	<.001	100
VIII	9	Had pain	.774	<.001	78.9
VIII	19	Pain interfered	.895	<.001	81.0
IX	8	Shortness of breath	.792	<.001	77.3
X	11	Sleep disturbance	.909	<.001	81.9
XI	13	Lack of appetite	.947	<.001	92.4
XII	16	Constipation	.899	<.001	66.6
XIII	17	Diarrhea	.965	<.001	94.0
XIV	28	Financial impact of disease	.922	<.001	85.8
XV	29	Physical condition	.830	<.001	70.0
XV	30	General QoL^a^	.863	<.001	65.0

^a^QoL: quality of life.

^b^statistically high significant correlations.

**Table 6:** Test of internal consistency in the function scales and symptom scales: results of Pearson correlation and Kendall tau analyses.

Scale		Pearson correlation (95% CI)	*P* value^a^	Kendall tau	*P* value^a^
**Functional scales**					
	Physical resilience	.979 (.966-.987)	<.001	.918	<.001
	Resilience at work and during leisure time activities	.900 (.851-.933)	<.001	.795	<.001
	Emotional resilience	.941 (.906-.963)	<.001	.848	<.001
	Cognitive resilience	.914 (.865-.945)	<.001	.866	<.001
	Social resilience	.921 (.874-.950)	<.001	.819	<.001
**Symptom scales**					
	Tiredness	.948 (.916-.968)	<.001	.851	<.001
	Nausea or vomiting	.956 (.928-.973)	<.001	.873	<.001
	Pain	.907 (.852-.942)	<.001	.819	<.001
	Shortness of breath	.798 (.712-.860)	<.001	.710	<.001
	Sleep disturbance	.921 (.874-.951)	<.001	.810	<.001
	Lack of appetite	.959 (.933-.974)	<.001	.916	<.001
	Constipation	.869 (.793-.917)	<.001	.828	<.001
	Diarrhea	.953 (.931-.968)	<.001	.928	<.001
	Financial impact of disease	.919 (.872-.949)	<.001	.863	<.001
	Overall state of health	.878 (.823-.916)	<.001	.769	<.001

^a^Statistically high significant correlations.

## Discussion

### Principal Findings

In both dimensions of reliability (parallel forms reliability and internal consistency), we found high correlations with only few differences in the patient’s response behavior between paper-based PRO and e-PRO in the EORTC QLQ-C30 questionnaire. In the test of parallel forms reliability, we found statistically significant differences in only 3 of 30 questions. By focalizing the function scales and the symptom scales, there was only one statistically significant difference between the patient’s answers in both procedures. In the dimension of consistency, there were high correlation and agreement rates in all items and scales. Due to only few differences and high correlations in almost all single items and scales, the PiiA tool’s e-PRO version of EORTC QLQ-C30 seems to be reliable for HRQoL measurement in patients with metastatic and adjuvant breast cancer. Due to the results, we cannot expect that the future use of the PiiA tool in the same patient group will show significant differences between paper-based PRO and e-PRO version of QLQ-C30, or that patient’ response behavior will be significantly influenced by the survey tool after transition to electronic-based patient surveys. Therefore, the tool is suitable for ascertaining HRQoL in patients with metastatic or adjuvant breast cancer.

### Comparison With Prior Work

Although e-PRO applications are on the rise, paper-based surveys of PRO still predominate clinical research, as there is a lack of reliable electronically validated questionnaires. One of the most used questionnaires for the measurement of HRQoL, especially in patients with breast cancer, is the EORTC QLQ-C30, with reliable paper-based format in many languages but without reliable electronic-based version in German. Electronic-based utilization of EORTC QLQ-C30 and other PRO without verification of reliability could endanger significance of e-PRO surveys, wherefore corresponding analysis in relation to differences and correlations between the paper-based vision of EORTC QLQ-C30 and newly developed online tools is of great importance. It can be assumed that several aspects (ie, sociodemographics, technical skills, health condition, and maybe design specifics of the e-PRO tool) influence the patient’s willingness to use e-PRO and their response behavior, which underlines the necessity of reliability analyses [41,42].

### Limitations

However, there were some limitations in the study design and the methodological implementation, which could possibly reduce data’s validity. In 3 questions of the test of parallel forms reliability, we found several missing values that could be because of the length of the survey. The patients were surveyed while they were receiving chemotherapy intervention, and they were not permitted to take the questionnaire home to complete it. Obviously, the length of the questionnaire had an effect on the patients’ concentration, as missing values were found especially in the second response run of the EORTC questionnaire. Possibly, the burden of therapy was potentially affecting the ability of some patients to fill both paper- and tablet-based questionnaires during an outpatient visit. Missing values were found particularly in the questions “need to rest” and “felt tired,” which were potentiated unfavorable to the dimension “tiredness” in the dimension of parallel forms reliability. In addition, the tumor stage, extent of metastasis, and the administered therapy were beyond the scope of study. Furthermore, psycho-oncological information was not collected, although psycho-oncological distress is a commonly associated burden that could potentially influence the willingness to use e-PRO and therefore e-PRO’s reliability [42,52]. It was not possible to determine whether the state of health was lower and the psychological distress was higher in those patients who could not be motivated to participate in the study compared with those who could be included, wherefore a selection bias was in place. Therefore, it is not possible to assess conclusively whether the e-PRO version is reliable for all patients with metastatic and adjuvant breast cancer or only for those with substantiated willingness. It must also be ensured that the proven reliability for the mentioned patient group applies only to the electronic-based version of the EORTC QLQ-C30 questionnaire but not to the PiiA tool per se.

### Strengths of the Study

The EORTC QLQ-C30 questionnaire has been used worldwide [45], but only with reliable paper-based versions, whereas e-PRO has become much more prevalent (and “user-friendly”) [46]. It is one of the strengths of this study that a new tool for e-PRO measurement was developed and analyzed regarding reliability in patients with breast cancer, while other studies often assign paper-based versions into a tablet-based format without verification of reliability. Reliability was ascertained in a multidimensional way, as parallel forms reliability (Wilcoxon test) and internal consistency (by focalizing Spearman rho, agreement rates, Pearson correlation and Kendall tau) were calculated. Our data show that patients with both the adjuvant and metastatic breast cancer are able to use e-PRO, as the PiiA tool was reliable in both patient groups. However, willingness to use e-PRO in patients with metastatic and adjuvant breast cancer is dependent on technical exposition [42]. The results of the study can improve the quality of e-PRO measurements, as they seem to be generalizable, and the PiiA application of EORTC QLQ-C30 can be used for reliable e-based measurement of HRQoL in other studies and clinical routine. The tool is reliable in female patients with breast cancer, as hurdles for e-PRO could be found especially in metastatic patients [42].

### Conclusions

Electronic-based PRO is constantly being adopted in clinical research and clinical routine, which underlines the necessity of reliable questionnaires. The evaluated PiiA’s version of the EORTC QLQ-C30 is reliable for patients with breast cancer in adjuvant or metastatic setting because high correlation was found in almost all questions (and in many scales). Thus, we conclude equality between the validated p-PRO assessment and the used e-PRO tool. However, the reliability in other prospective trials should also be analyzed to ensure the reliable usability in all patient groups.

## Abbreviations

CONSORT: Consolidated Standards of Reporting Trials
EORTC QLQ‑C30: European Organization for Research and Treatment of Cancer Quality of Life Questionnaire-Core 30 item
e-PRO: electronic-based patient-reported outcome
e-PROCOM: Patient-Reported Outcomes and Compliance Analysis
FACT-G: Functional Assessment of Cancer Therapy-General
HRQoL: health-related quality of life
MD: median
PRO: patient-reported outcome
QoL: quality of life
SD: standard deviation
SPSS: Statistical Package for the Social Sciences version

## Appendix

Multimedia Appendix 1 CONSORT-EHEALTH checklist (v1.6.1).
